# Pandora’s Box

**DOI:** 10.1192/bji.2025.10085

**Published:** 2026-02

## Can we tell how fast we are ageing?

The subject has been of interest to both the scientific and consumer worlds, and various blood indices have been studied for some time. A recently published study from Duke University in Durham, North Carolina, USA, carried out brain magnetic resonance imaging scans of 1000 people, born in Dunedin, New Zealand, from April 1972 to March 1973, who had periodically undergone various assessments as they were growing up. The authors used 860 brain images and examined correlations with participants’ age-related decline in cardiovascular, metabolic and immune function and other physiological measures. They produced a new ‘ageing clock’ and found that higher scores were correlated with greater risk of future chronic disease and death. Notably, the ageing clock showed the same results when the researchers used images from the UK Biobank (42 583 participants) and the Alzheimer’s Disease Neuroimaging Initiative (1737 participants).

The authors recognise that a lot more data in more populations and different age groups are needed to test how to harmonise data from different types of scanner before the clock can be considered for use in clinical settings. In the meantime, they are also interested in whether conditions such as schizophrenia or sleep disorders maybe associated with accelerated ageing.

**Whitman ET, Elliott ML, Knodt AR, Abraham WC, Anderson TJ, Cutfield NJ, Hogan S, Ireland D, Melzer TR, Ramrakha S, Sugden K, Theodore R, Williams BS, Caspi A, Moffitt TE, Hariri AR.** DunedinPACNI estimates the longitudinal Pace of Aging from a single brain image to track health and disease. *Nat Aging*. 2025 Aug; **5**(8): 1619–1636. doi: 10.1038/s43587-025-00897-z. Epub 2025 Jul 1. PMID: 40595015; PMCID: PMC12350157.

## Learn another language and keep your brain young

While we are waiting for the ageing clock to predict our future health and survival, what can we do to improve our chances? A recent study highlighted the protective value of multilingualism on ageing. The researchers developed a biobehavioural model quantifying ageing rates (delayed or accelerated) in a large number of individuals (*n* = 86 149) from 27 European countries. They obtained information from national surveys on positive factors such as functional ability, education, cognition, as well as adverse factors such as cardiac and metabolic conditions, sensory impairments and also female sex. Multilingualism at country level was an aggregate exposure.

They found that positive biobehavioural factors were linked to delayed ageing, whereas adverse factors were linked to accelerated ageing. Monolingualism was associated with increased risk of ageing, whereas multilingualism had a protective role both cross-sectionally and in longitudinal analyses. This protective effect was not affected by linguistic or sociopolitical factors.

For your health’s sake, get on with learning another language. It may be more interesting and useful than doing the crossword.

**Amoruso L, Hernandez H, Santamaria-Garcia H, Moguilner S, Legaz A, Prado P,** et al. Multilingualism protects against accelerated aging in cross-sectional and longitudinal analyses of 27 European countries. *Nat Aging* 2025; **5**: 2340–54.

## Don’t be a ‘debutante’ author

It is common, following the publication of a paper, for somebody else to write a letter raising criticisms. In a similar situation after the publication of their paper on malaria in the *New England Journal of Medicine*, Carlos Chaccour and Matthew Rudd observed that the letter cited some of their previous publications that did not support its claims. Wary of artificial intelligence involvement, they suspected that the letter was written by a machine.

Following this, they looked into more than 730 000 letters published in the past 20 years and found that from 2023 to 2025 there was a small group of what they called ‘prolific debutante authors’ who appeared in the top 5% of letter writers and had used programs such as ChatGPT and artificial intelligence chatbots. Their letters appeared in 1930 journals, including 175 in the *Lancet* and 122 in the *New England Journal of Medicine*. Other studies also documented an increase in research articles that had signs of artificial-intelligence-written text.

This was considered an unscrupulous way of letter authors padding their CVs at the expense of letters sent by others. Normally, letters are not subject to peer review. References used in the letters were artificial intelligence produced, and were often incorrect and contained errors that could mislead readers. They could also damage the reputation of authors, who may have spent years on a piece of research before it came to publication.

How can this practice be controlled? Journal editors need to scrutinise submissions of letters with the same vigour as paper submissions.

**Brainard J.** Letters to scientific journals surge as ‘prolific debutante’ authors likely use AI. *Science* 2025. Available from: https://doi.org/10.1126/science.z0lg2zx:10.1126/science.z01g2zx.

## Artificial intelligence both a friend and a foe in research

Major scientific journals and conferences do not credit artificial intelligence programs such as ChatGPT as either authors or reviewers of studies, as it is accepted that computers cannot be held accountable. However, a change may be on the horizon. A recent meeting called Agents4Science may be changing the rules. The aim of the conference was to advance ‘the development of guidelines for responsible artificial intelligence participation in science’. The organisers’ hope was that this could accelerate science and ease the burden on peer reviewers, given the huge number of submissions to journals and conferences. Others, however, are very critical of this premise. Raffaele Ciriello, a digital innovation researcher from the University of Sydney, released a statement through the Science Media Centre before the meeting stating that ‘no human should mistake this for scholarship’, and that ‘science is not a factory that converts data into conclusions […] Treating research as a mechanistic pipeline … presumes that the process of inquiry is irrelevant so long as the outputs appear statistically valid’. The conference organisers disagreed. They had 315 papers submitted, reviewed and scored on a six-point scale by three language models, GPT-5, Gemini 2.4 Pro and Claude Sonnet 4. The mean score ranged from 2.3 to 4.2. Humans were also asked to review 80 papers that passed the threshold score, and the organisers accepted 48 based on both the artificial intelligence and human reviews. The subjects of the papers included chemistry, medicine and psychology.

The enthusiasm was tempered by the observation that the existing large language models have so far shown a ‘sycophantic’ tendency to produce outputs that reflect favourably a human request. It was also noted that they are ‘not going to produce the level of conflict and diverse perspectives that are required for really pathbreaking work’.

**Brainard J.** At futuristic meeting, AIs took the lead in producing and reviewing all the studies. *Science* 2025. Available from: https://doi.org/10.1126/science.zp11qde.

